# ICSI with testicular sperm for couples with sperm DNA damage

**DOI:** 10.1590/S1677-5538.IBJU.2018.04.02

**Published:** 2018

**Authors:** Armand Zini

**Affiliations:** 1 Department of Surgery, St. Mary's Hospital, McGill University, Montreal, Canada

The advent of intracytoplasmic sperm injection (ICSI) has revolutionized the management of male factor infertility (1). Shortly after the technique was introduced, studies demonstrated that ICSI could successfully treat couples with severe male factor infertility. Several investigators reported that neither sperm concentration, morphology, nor progressive motility had any impact on ICSI outcomes (2-4). However, the only sperm characteristic that portended a negative ICSI outcome was the injection of a totally immotile (and presumably dead) spermatozoon (4-6).

More recent publications have shown that ICSI may not overcome significant sperm abnormalities. Mitchell et al. reported significantly lower clinical pregnancy rates in couples with sperm motility <5% compared to those with higher sperm motility (11% vs. 41%, P=0.04 for couples with sperm motility <5% and >5%, respectively) (7). De Vos et al. reported significantly higher clinical pregnancy (37% vs. 20%, respectively, P=0.018), implantation (32% vs. 23%, respectively, P=0.013) and live birth rates (28% vs. 20%, respectively, P=0.006) with the use of morphologically normal vs. morphologically abnormal sperm for ICSI (8). Strassburger et al. studied 1,076 unselected ICSI cycles and reported that cryptozoospermic couples had significantly lower fertilization and clinical pregnancy rates (46% vs. 61%, P<0.0001 and 20% vs. 31%, P<0.05, respectively) and higher miscarriage rates (30% vs. 15%, P<0.03) when compared to couples with a sperm concentration between 1×10^5^ sperm/mL and 1×10^7^ sperm/mL (9).

Subfertile men with abnormal semen parameters may have an underlying sperm genetic defect that could potentially impact on IVF and ICSI outcomes. Indeed, studies have shown that sperm DNA damage is more common in men with poor semen parameters than in those with normal parameters and in 2008 a systematic review and meta-analysis of 2,162 IVF and ICSI treatment cycles demonstrated a potential adverse effect of sperm DNA damage on the chance of pregnancy, with a diagnostic odds ratio of 1.44 (95% CI, 1.03, 2.03) (10, 11). Moreover, a study of 1,549 IVF/ICSI cycles concluded that sperm DNA damage was predictive of pregnancy loss after IVF/ICSI (combined OR 2.48; 95% CI 1.524.04; P<0.0001) (12). More recent meta-analyses have similarly reported that sperm DNA damage is associated with lower clinical pregnancy rates and higher miscarriage rates after IVF and ICSI (13-15).

In 2005, Greco et al., reported their experience with the use of testicular sperm with ICSI in a small series of couples with sperm DNA damage (16). Greco et al., claimed that there was “no specific treatment” for sperm DNA damage and hypothesized that the DNA damage in ejaculated sperm begins after spermatozoa are released from Sertoli cells. In view of the adverse impact of sperm DNA damage on IVF and ICSI outcomes, Greco et al. proposed using testicular sperm in men with sperm DNA damage with the idea that sperm recovered directly from the testis would show less damage than ejaculated sperm. To test this idea or hypothesis, they (1) compared the DNA damage in ejaculated and testicular sperm in two sequential assisted reproduction cycles performed in couples with high levels of ejaculated sperm DNA damage and (2) evaluated the ICSI outcomes of these couples. Greco et al. reported higher pregnancy rates with ICSI when using testicular rather than ejaculated sperm in couples with sperm DNA damage and observed a higher frequency of sperm showing detectable DNA damage in ejaculated vs. testicular sperm (16). They suggested that the poorer outcome with ejaculated sperm was a result of acquired DNA damage during transit through the epididymis or possibly during ejaculation.

In 2005, Suganuma et al. conducted experimental studies using an animal model with abnormal spermatogenesis (mutant mice with minimal levels of transition nuclear proteins and incomplete sperm nuclear compaction). They planned to investigate whether DNA damage in ejaculated sperm is increased after spermatozoa are released from the testis (17). Suganuma et al. observed that in animals with abnormal spermatogenesis the passage of sperm through the epididymis was associated with a loss of sperm DNA integrity and fertilizing capacity (17). They speculated that in animals with poor sperm nuclear compaction, the sperm DNA is not fully protected during epididymal passage. In contrast, in animals with normal spermatogenesis, the passage of sperm through the epididymis was not associated with a similar loss of sperm DNA integrity and fertilizing capacity. As such, Suganuma et al. proposed that in some men (i.e. those with defective spermatogenesis) the passage of sperm through the epididymis could result in a loss of sperm DNA integrity and fertilizing capacity. This concept (adverse effect of epididymal transit on sperm nuclear integrity) is contrary to the understanding of the biology of sperm maturation, specifically, the acquisition of sperm motility and nuclear compaction during passage of spermatozoa through the epididymis (18, 19).

The idea that the post-testicular environment or epididymal transit can induce sperm damage has led clinicians to utilize testicular rather than ejaculated sperm for ICSI in men with abnormal spermatogenesis and poor sperm DNA integrity. In a recent online survey of Canadian fertility clinics, 70% of the respondents reported performing testicular sperm retrieval (TSR) with ICSI for non-azoospermic men with poor sperm DNA integrity (Zini et al., unpublished observations). Similarly, over 70% of the respondents attending a session on testicular sperm for ICSI at the 2017 annual meeting of the American Society for Reproductive Medicine reported that they would (in selected cases) opt for testicular sperm rather than ejaculated sperm ICSI in men with sperm DNA damage (Zini et al., unpublished observations). However, despite the widespread utilization of TSR with ICSI in these men, there is no consensus or guideline on how to manage these cases in clinical practice.

In this issue of the International Brazilian Journal of Urology, Dr. Sandro Esteves and Dr. Mark Sigman present the Pro and Con perspectives, respectively, of using testicular rather than ejaculated sperm in non-azoospermic couples with high sperm DNA fragmentation (20, 21). Dr. Esteves discusses the adverse impact of sperm DNA damage on reproductive outcomes and demonstrates that sperm DNA damage is lower in testicular than in ejaculated sperm. Dr. Esteves presents a systematic of the literature and provides us with compelling evidence in support of the use of testicular rather than ejaculated sperm in these couples. Dr. Sigman argues that the quality of the studies on testicular sperm-ICSI is moderate at best and that there is insufficient evidence to adopt the practice of testicular sperm retrieval in these couples. Moreover, Dr. Sigman also cautions that sperm DNA testing requires further validation as a diagnostic test and in this context as well.
